# Selection of optimal reference genes for normalization in quantitative RT-PCR

**DOI:** 10.1186/1471-2105-11-253

**Published:** 2010-05-14

**Authors:** Inna Chervoneva, Yanyan Li, Stephanie Schulz, Sean Croker, Chantell Wilson, Scott A Waldman, Terry Hyslop

**Affiliations:** 1 Department of Pharmacology and Experimental Therapeutics, Thomas Jefferson University, Philadelphia, PA 19107, USA; 2 Respiratory Research, Teva Branded Pharmaceutical Products R&D, Inc., Horsham, PA 19044, USA; 3 Department of Experimental Medicine, Proctor and Gamble, Cincinnati, OH 45241, USA

## Abstract

**Background:**

Normalization in real-time qRT-PCR is necessary to compensate for experimental variation. A popular normalization strategy employs reference gene(s), which may introduce additional variability into normalized expression levels due to innate variation (between tissues, individuals, etc). To minimize this innate variability, multiple reference genes are used. Current methods of selecting reference genes make an assumption of independence in their innate variation. This assumption is not always justified, which may lead to selecting a suboptimal set of reference genes.

**Results:**

We propose a robust approach for selecting optimal subset(s) of reference genes with the smallest variance of the corresponding normalizing factors. The normalizing factor variance estimates are based on the estimated unstructured covariance matrix of all available candidate reference genes, adjusting for all possible correlations. Robustness is achieved through bootstrapping all candidate reference gene data and obtaining the bootstrap upper confidence limits for the variances of the log-transformed normalizing factors. The selection of the reference gene subset is optimized with respect to one of the following criteria: (A) to minimize the variability of the normalizing factor; (B) to minimize the number of reference genes with acceptable upper limit on variability of the normalizing factor, (C) to minimize the average rank of the variance of the normalizing factor. The proposed approach evaluates all gene subsets of various sizes rather than ranking individual reference genes by their stability, as in the previous work. In two publicly available data sets and one new data set, our approach identified subset(s) of reference genes with smaller empirical variance of the normalizing factor than in subsets identified using previously published methods. A small simulation study indicated an advantage of the proposed approach in terms of sensitivity to identify the true optimal reference subset in the presence of even modest, especially negative correlation among the candidate reference genes.

**Conclusions:**

The proposed approach performs comprehensive and robust evaluation of the variability of normalizing factors based on all possible subsets of candidate reference genes. The results of this evaluation provide flexibility to choose from important criteria for selecting the optimal subset(s) of reference genes, unless one subset meets all the criteria. This approach identifies gene subset(s) with smaller variability of normalizing factors than current standard approaches, particularly if there is some nontrivial innate correlation among the candidate genes.

## Background

Normalization is important in real-time qRT-PCR analysis because of the need to compensate for intra- and inter-kinetic RT-PCR variations [1-3]. Such variations may be due, for example, to the difference in amount of starting material between the samples, difference in RNA integrity, cDNA sample loading variation, or difference in RT efficiency. One of the most popular methods is normalizing a target gene expression to the ribosomal RNAs (rRNA) or messenger RNAs (mRNA) from an internal control or reference gene(s). Such reference genes, also called housekeeping genes, should be expressed in abundance, not be co-regulated with the target gene, and have minimal innate variability. On the other hand, the expression of these genes should vary in accordance with the experimental error associated with the technique (due to sample processing and loading, etc) in order to correct for these errors through normalization.

The variability of a reference gene has two major sources, experimental variability associated with the technology and the innate or natural variability of the reference gene (between tissues, individuals, etc). The original approach to normalization was to find a single reference gene with the most stable (in the sense of the smallest variability) expression across tissues and individuals. Starting with the work of Vandesompele et al [4], normalization is carried out using a geometric mean (inverse natural logarithm of the mean of the log-transformed gene expressions) of multiple internal control genes as a normalizing factor. The rationale is that the same experimental error should be present in all genes expressed in the same sample, if all genes are processed simultaneously. Thus, the experimental errors of individual replicates are averaged across the reference genes, and a geometric mean provides a more robust estimate of the experimental error than individual reference genes. In cases of unregulated and uncorrelated reference genes, the innate variance component of the geometric mean variance is no larger than the largest innate variance component of individual reference genes divided by their total number. Therefore, by increasing the number of reference genes with bounded innate variance, one can theoretically make the innate variance of their geometric mean as small as desired. However, it is expensive and impractical to process too many reference genes for each sample. Thus, careful selection of a small reference genes subset with optimal properties is very important.

It is well documented that optimal reference genes vary according to tissues and treatments [5-7] and that the final choice of the reference genes should be validated for each particular qRT-PCR study [1,2,6]. Thus, as a part of assay validation, candidate reference genes are studied and optimal genes selected for inclusion into normalizing factors.

Vandesompele et al [4] proposed an algorithm that ranks individual candidate reference genes according to their stability measure, which is the average pairwise variation of a particular gene with all other candidate reference genes. The pairwise variation is defined as the standard deviation of the log-transformed ratios of expressions of paired genes. The algorithm first selects a pair of two candidate reference genes that have the highest expression agreement (that is the smallest variability in the ratios) among all possible pairs of genes. Then, the next stable reference gene is identified as the one, which has the highest agreement with the rest of the candidate genes and with the geometric mean of the first two selected reference genes, and so on. Thus, the algorithm relies on sequential pair-wise comparisons, which does not guarantee that the optimal subset of three or more genes would be identified.

A more comprehensive approach to selection of the optimal subset of reference genes is to fit a common model that would allow simultaneous quantification and comparison of variability in all candidate genes. This is the approach taken, for example, in [8-10], where various ANOVA and linear mixed effects models were used for the log-transformed gene expression ratios of all candidate reference genes at once. These models incorporate the average gene effect or the average gene-by-tissue type effect (if multiple tissue types are considered), the effect of each individual sample (within each tissue type) and heteroscedastic error terms with the variances that differ by gene and tissue type. Szabo et al [9] used the variance component estimates from the model to rank the variances of the candidate reference genes and estimate the standard deviation of the log geometric means of the best (in the sense of the smallest variability) gene set for each possible set size (1, 2, 3, and so on). Andersen et al [8] proposed a new measure of gene expression stability based on the variance components estimates from the fitted ANOVA model. Similar to [4], this stability measure also allows ranking individual candidate reference genes from the most to the least stable. Abruzzo et al [10] considered linear mixed effects models for log-transformed gene expression and demonstrated that treating experimental errors as random effects provides a much better model fit than using ANOVA models, whose assumptions were violated.

The crucial assumption underlying all these methods is independence in innate variation of the candidate reference genes. The corresponding statistical models assume that correlation between expressions of different genes in the same sample comes exclusively from the experimental variation in the sample. In contrast, we have observed that even after subtracting the random (or fixed) effects of sample, residuals may exhibit non-trivial correlation between some candidate reference genes (see Results). Therefore, estimates of the standard deviation of the log geometric mean may change substantially when correlation is properly estimated and incorporated. This, in turn, can change the ranking of a subset of candidate reference genes with respect to optimality for inclusion into normalization factors.

We developed a robust approach for directly selecting optimal subset(s) of reference genes rather than addressing stability of individual candidate genes. Our approach is based on estimating the unstructured covariance matrix of all available candidate reference genes and using this covariance matrix to estimate the variances of the log normalizing factors (geometric means of the expression of multiple genes) corresponding to all possible subsets of reference genes. Robustness is achieved through bootstrapping candidate reference gene samples and obtaining the bootstrap upper confidence limits for the variances of the log transformed normalizing factors and average ranks of reference gene subsets with respect to the variance of their geometric mean in all bootstrap samples. A bootstrap procedure was proposed earlier [11] to maximize the robustness of the approach in [4] for ranking individual genes. In contrast, our procedure ranks the entire gene subsets of all possible sizes. Using the proposed approach, the optimal subset of the reference genes may be selected (A) to minimize the variability of the normalizing factor; (B) to minimize the number of reference genes with acceptable upper limit on variability of the normalizing factor; or (C) to minimize the average rank of the variance of the normalizing factor.

Two publicly available data sets and one new data set from the validation study of five candidate reference genes for normalization of guanylyl cyclase C (GUCY2C) mRNA expression in blood are used to illustrate the proposed method and compare to earlier published results. In addition, a small simulation study was conducted to evaluate the performance of the proposed approach under known correlation structures assuming varying degrees of innate correlation among candidate reference genes.

## Methods

### Model for the log-transformed expression levels of candidate reference genes

To incorporate all correlations among candidate reference genes, we simultaneously model their log-transformed expression levels or threshold cycle (Ct) numbers in a multivariate linear mixed effects model with unstructured covariance matrix. The normality assumption is usually appropriate for log-transformed expression levels or Ct numbers in homogeneous populations of samples.

Let *y*_*jik *_be the *k*th, *k *= 1,..., K, replicate of the log-transformed expression level or threshold cycle Ct for the candidate reference gene *j*, *j *= 1,..., J, in sample *i, i *= 1,..., N. Denote by **Y**_*ik *_= [*y*_*1ik*_,..., *y*_*Jik*_]^T ^the vector of log-transformed expression levels for all J candidate reference genes in replicate *k *of sample *i*. For a homogeneous population of samples, vector **Y**_*ik *_may be modeled as(1)

where vector **g **= [*g*_*1*_,...,g_*J*_] ^T ^and g_*j *_is the average log-transformed expression level for the candidate reference gene *j*, **s**_*i *_= [s_*i*_,..., s_*i*_] ^T ^is the random effect of *i*^th ^sample, which reflects the experimental variation and is the same for all genes, so that **s**_*i *_= s_*i *_[1,...,1] ^T^, **r**_*i *_= [r_*i1 *_,..., r_*iJ *_] ^T ^is the vector of random gene effects in sample *i*, and **e**_*ik *_= [e_*ik1 *_,..., e_*ikJ *_] ^T ^is the vector of error terms in replicate k.

It is assumed that sample random effects s_*i*_, random gene effects vectors **r**_*i*_, and the error terms vector **e**_*ik *_are all independent, s_*i *_are identically normally distributed as N(0, σ^2^), vectors **r**_*i *_are identically normally distributed as *J*-variate normal distribution MVN_*J*_(0,**R**), and **e**_*ik *_are identically distributed as MVN_*J*_(0,**D**), **D **= *Diag(τ*_*1*_^*2*^,..., *τ*_*J*_^*2*^). For each gene *j *and sample *i*, model (1) implies that

and vectors **Y**_*ik *_have a multivariate normal distribution(2)

where **V **= σ^2^1_J×J _+ **R **+ **D **and 1_J×J _is J×J matrix of ones.

Our model (1) generalizes models 4 and 5 in [10] by assuming a general unstructured positive definite matrix **R **rather than imposing a simple uncorrelated structure with **R **= *Diag(δ*_*1*_^*2*^,..., *δ*_*J*_^*2*^). Sunberg et al [12] also mention in discussion a model similar to (1) in terms of covariance structure.

For multiple tissues and possible covariates affecting **Y**_*ik *_the mean vector **g **would have to be replaced by some linear mean model. Since the proposed methodology utilizes only covariance parameters estimates, it is straightforward to extend developments to the case with a linear mean model instead of the mean vector **g**. The standard way to write a general linear mean model is **Aβ**, where A is some design matrix and **β **is the vector of unknown parameters. For model (1), **A **is just the identity matrix and **β = g**. In the general case, the model is written as(3)

Notably, such extension has no effect on the assumed covariance structure of the data. For example with just multiple tissues, t = 1,.., T, one can use the model

where vector **g **= [*g*_*1*_,...,g_*J*_] ^T ^represents now across tissues average log-transformed expression levels for all candidate reference genes *j *= 1,..., J, and vector **g**_t _represents the mean differences in expression attributed to tissue *t*.

In most analyses of qRT-PCR data, the Ct numbers for the replicates of the same reaction are averaged, and the majority of methods for selecting optimal subsets of reference genes also operate with averaged replicates, which is appropriate if averaged replicates are to be used for normalizing the target gene. For this reason, and to simplify notation, in further development we do not use multiple replicates of the same reaction. With averaged replicates, vectors **Y**_*ik *_and **e**_*ik *_in model (1) no longer depend on index k and model (1) is simplified to:(4)

where vectors **r**_*i *_effectively incorporate both, the random gene effects and the errors of gene expression measures. The multivariate formulation (2) still applies to model (4) with **V **= σ^2^1_J×J _+ **R**. If we consider a specific case of model (4) with s_i _being fixed rather than random effects (so that **V = R**) and **R **= *Diag(δ*_*1*_^*2*^,..., *δ*_*J*_^*2*^) then we obtain model 1a in [9].

In general, the variance components σ^2^1_J×J_, **R**, and **D **in models (1) and (4) are not identifiable unless one imposes additional constraints on the structure of **R **and **D**. In previous work, **R **was constrained to be diagonal, which corresponds to the independent random effects of reference genes. Our approach is to estimate **V **as an unstructured covariance matrix without separating the variance components, and then use **V **to compute the variance of the log geometric mean of any possible subset of reference genes. An unstructured J×J matrix **V **has J(J + 1)/2 unknown parameters, with the total of J(J + 1)/2 + J = J(J + 3)/2 unknown parameters for model (2). Hence, one needs at least samples of size N > (J + 3)/2 to estimate model (2). With a moderate number of samples available, the estimates of **V **may not be reliable. To overcome this, we propose to utilize bootstrap re-sampling and compute the upper confidence bounds for the variances of the geometric means. Such upper confidence bounds would properly reflect uncertainty in estimation of the variances.

### Variability of geometric means of multiple genes

Further we focus on single or averaged multiple replicates of a gene in the sample and assume model (4) with **V **= σ^2^1_J×J _+ **R**. The log geometric mean expression of a subset of *L *≤ *J *reference genes j_1_, j_2 _,...,j_L _in sample *i *is computed as(5)

In a vector form, (5) may be written as

where *J*x*1 *vector *C*_*j1,...,jL *_has elements equal to 1, if *j = j*_*1*_, *j*_*2 *_,..., *j*_*L*_, and elements equal to 0 otherwise. Since **Y**_*i *_= MVN_*J *_(**g**, **V**), the variance of F_*i*_(*j*_*1*_,..., *j*_*L*_) is(6)

Thus, the total variance of the log geometric mean of any subset *j*_*1*_, *j*_*2*_,..., *j*_*L *_of reference genes may be estimated using (6) with the corresponding vector *C*_*j1,...,jL *_and matrix **V**, which is estimated by fitting model **Y**_*i *_~MVN_*J *_(**g**, **V**). Representation (6) allows computing the variance of all possible F_*i*_(*j*_*1*_,..., *j*_*L*_) through the nested J cycles exhausting all possibilities for vectors *C*_*j1,...,jL*_.

When **V **= σ^2^1_J×J _+ **R**, then (6) implies(7)

Hence, the log geometric mean of any subset of reference genes includes the same variance component σ^2 ^corresponding to the experimental error present in all gene expressions for the same sample. Therefore, minimizing the total variability of the log geometric mean is equivalent to minimizing the variability described by **R**.

### Selection of the optimal subset of reference genes

Using model (4) and expression (6), we propose a robust approach for selecting optimal subset(s) of reference genes with the smallest variance of the corresponding normalizing factors. Robustness is achieved through bootstrapping candidate reference genes data to obtain the bootstrap upper confidence limits for the variances of the (log) normalizing factors (geometric means) for all possible gene subsets as well as the distribution of ranks of these variances. The bootstrapping also alleviates the uncertainty in estimation of potentially large number of parameters in unstructured covariance matrix **V**.

Specifically, for each bootstrap sample, the following analyses are performed:

(i) Unstructured covariance matrix **V **of all available candidate reference genes is estimated from model (2). In this work, the estimates of V were computed in SAS PROC MIXED (SAS 9.2, SAS Institute, Cary, NC), but any other software capable of fitting liner mixed effects or MANOVA models may be used as well.

(ii) Vectors C_j1,...,jL _for all possible subsets of reference genes are generated and expression (6) is used to compute the variance of the log geometric mean for each possible subset of reference genes. There is a finite, although rather large number, 2^J^-1, of possible subsets of J reference genes, and the absolute minimum is always attained. In practical qRT-PCR validation studies, the number of candidate reference genes J would not be expected to be much larger than 10.

(iii) All possible subsets of reference genes are ranked from the smallest to the largest variance of the corresponding log geometric mean.

Based on results for all bootstrap samples, we compute the bootstrapped upper 95% confidence limit for the variance of the log geometric mean and the average rank of this variance for all possible subsets of the reference genes. Then the optimal subset of the reference genes may be selected using one of the following criteria:

**(A) **to minimize the upper 95% confidence limit on variability of the log geometric mean regardless of the number of reference genes required;

**(B) **to minimize the number of reference genes given that the upper 95% confidence limit on variability is under some acceptable level;

**(C) **to minimize the average rank of the variance of the log geometric mean.

**Figure 1:**
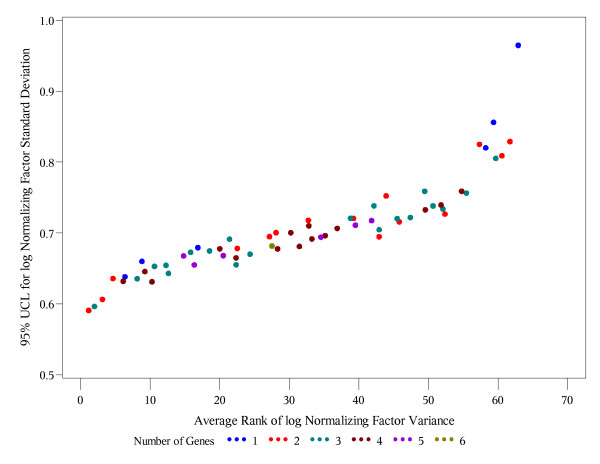
**Breast tumor data: 95% UCL vs. the average overall rank of the normalizing factors**. Each point represents one of the possible 63 = 2^6^-1 gene subsets. Different colors are used for the subsets with different numbers of genes included. The x-coordinate is the average overall rank of the corresponding normalizing factor variance. The y-coordinate is the upper 95% confidence limit (95% UCL) for the standard deviation of the log normalizing factor. The red dot, which is closest to the lower left corner, represents the optimal (in the sense of criteria A and C) combination of two genes, ACTB and SF3A1.

**Figure 2:**
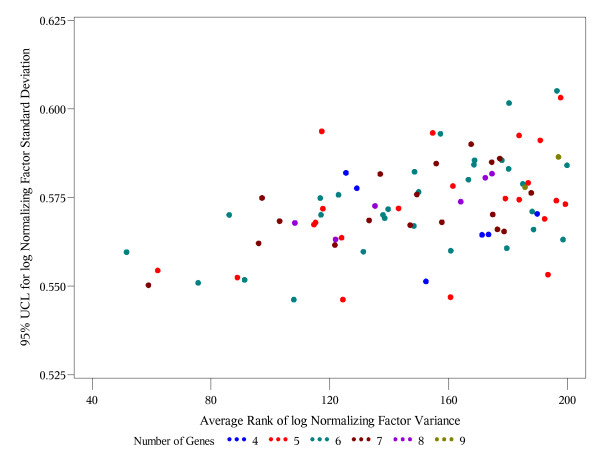
**Neuroblastoma data: 95% UCL vs. the average overall rank of the normalizing factors**. Each point represents one of the possible 1023 = 2^10^-1 gene subsets. Different colors are used for the subsets with different numbers of genes included. The x-coordinate is the average overall rank of the corresponding normalizing factor variance. The y-coordinate is the upper 95% confidence limit (95% UCL) for the standard deviation of the log normalizing factor. Only sets with average rank less than 200 are shown on the plot.

**Figure 3:**
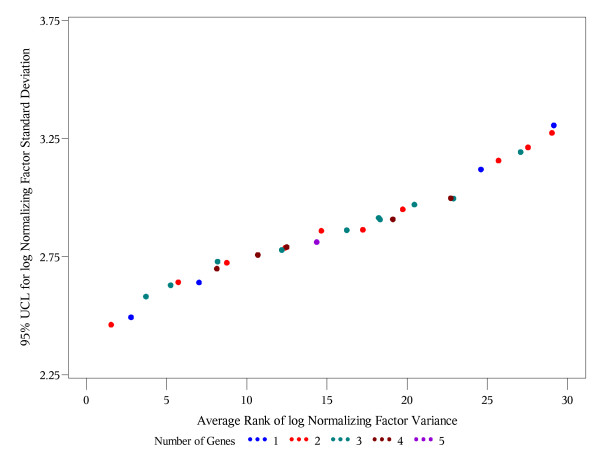
**Blood data: 95% UCL vs. the average overall rank of the normalizing factors (Ct numbers)**. Each point represents one of the possible 33 = 2^5^-1 gene subsets. Different colors are used for the subsets with different numbers of genes included. The x-coordinate is the average overall rank of the corresponding normalizing factor variance. The y-coordinate is the upper 95% confidence limit (95% UCL) for the standard deviation of the log normalizing factor.

The last criterion is similar in spirit to the bootstrap ranking procedure in [11], with an essential difference that they rank individual genes using the approach in [4], while our procedure ranks the entire gene subsets of all possible sizes. Also, rather than considering the entire distribution of ranks, which is cumbersome for (2^J^-1) possible subsets instead of just J reference genes, we use the mean rank (average in all bootstrap samples) as the measure of optimality in criterion (C). In the absence of a desired limit on variability in criterion (B), one ideally would want to find a reference gene subset that satisfies both, criteria (A) and (C). To address criteria (A) and (C) simultaneously, we plot the upper 95% confidence limits vs. the average rank by the size of gene subset (Figures 1, 2, 3). Such plots help to evaluate how far or how close the competing gene subsets are in terms of both criteria. A subset which is closest to the lower left corner is optimal using both criteria (A) and (C). If more than one subset is approximately the same distance from the lower left corner (Figure 3), then it is reasonable to pick the one with the smaller number of genes as an optimal.

A simple direct comparison of our method vs. previously proposed methods was performed by computing the log geometric mean and its variance for the optimal subsets (for each set size) selected by different procedures. The advantage is direct evaluation of the log geometric mean of interest while ignoring the rest of the genes, which mimics the prospective use of the selected reference genes (the other candidate reference genes would not be available).

The macros implementing the proposed methodology were developed in SAS 9.2 (SAS Institute, Cary, NC). The corresponding SAS code is included as Additional file 1. Simulation study was also performed using SAS 9.2. The real data were analysed in SAS 9.2 and geNorm 3.5 (VBA applet for Microsoft Excel 2000/XP/2003, version 3.5 http://medgen.ugent.be/~jvdesomp/genorm/).

## Results

### Data Sets

The first dataset includes relative expression levels of 6 reference genes (ACTB, GAPDH, MRPL19, PSMC4, PUM1, and SF3A1) quantified in 80 breast tumor samples. These data are described in detail in [9] and available for download http://genomebiology.com/content/supplementary/gb-2004-5-8-r59-s1.xls. The second dataset includes expression levels for 10 reference genes ACTB, B2M, GAPDH, HMBS, HPRTI, RPLI3A, SDHA, TBP, UBC, YWHAZ) quantified in 37 neuroblastoma samples. These data (available at the web site http://genomebiology.com/2002/3/7/RESEARCH/0034/additional/) are part of the data from various tissues that were used and described in [4]. This subset was selected because the number of neuroblastoma samples (37) was the highest among all tissue types included in the study reported in [4].

The third dataset comes from a validation study of five candidate reference genes for normalization of guanylyl cyclase C (GUCY2C) mRNA expression in blood. The RT-PCR assay to quantify GUCY2C mRNA in tissues and blood employing external calibration standards of RNA complementary to GUCY2C (cRNA) is described in [13]. This work is a part of the ongoing multi-institutional NCI-funded study of GUCY2C as a biomarker for colorectal cancer [14]. The study will determine the utility of GUCY2C mRNA expression in blood for early detection of recurrence in patients with colorectal cancer. Five candidate reference genes for normalization of GUCY2C expression include ACTB, glyceraldehyde-3-phosphate dehydrogenase (GAPDH), transferrin receptor (TFRC), peptidylprolyl isomerase B (PPIB), and hypoxanthine-guanine phosphoribosyltransferase (HPRT). These genes were previously considered as candidate reference genes for normalizing mRNA expression of various targets in blood. RT-PCR experiments were conducted using an ABI 7900 Sequence Detection System (Applied Biosystems, Foster City, CA). Blood samples from 25 healthy volunteers were analyzed as a part of the validation study of five candidate reference genes. Blood was collected in PaxGene Blood RNA tubes (Qiagen), and RNA was purified according to the manufacturer's instructions.

Here, the log transformed expression levels were computed from the threshold cycle (Ct) numbers as in the MS Excel add-on software gNorm, which implements the method described in [4]. For each candidate reference gene, the largest Ct number is subtracted from the Ct number for each sample, and the difference is exponentiated with the base 2. The resulting expression levels range between 0 and 1, with 1 corresponding to the sample with the smallest threshold cycle number and presumably the largest copy number of the corresponding reference gene.

### Results for the breast tumour data

In the breast tumour data, we first investigated innate correlation among 6 reference genes using the residuals from model 1a in [9]:(8)

where s_*i *_are assumed to be fixed rather than random effects and each gene is assumed to have different variance, *y*_*ji *_~N (g_j_, *τ*_*j*_^*2*^). The residuals were computed as(9)

where  and  are estimated by fitting model (8). Since s_*i *_represents experimental variability in *y*_*ji *_common for all reference genes in sample *i*, the residuals  represent only the innate between-individual variability. Table 1 presents the Pearson correlation matrix of residuals (9) from model (8) fitted to the data from 80 breast tumor samples. Note that the Pearson correlation coefficient is significantly different from zero for seven pairs of genes (in bold). Hence, the variance of the geometric means should depend not only on the variances of the corresponding genes, but also on their correlation, which cannot be ignored.

**Table 1:** Pearson correlation matrix of the residuals from model (8) fitted to the data from 80 breast tumor samples

		ACTB	GAPDH	MRPL	PSMC4	PUM
GAPDH	Coeff.^1^	-0.112				
	p-value	0.324				
MRPL	Coeff.^1^	**-0.476**	0.021			
	p-value	<.0001	0.851			
PSMC4	Coeff.^1^	**-0.246**	-0.108	0.014		
	p-value	0.028	0.340	0.903		
PUM	Coeff.^1^	0.077	**-0.352**	**-0.432**	**-0.510**	
	p-value	0.496	0.001	<.0001	<.0001	
SF3A1	Coeff.^1^	0.147	-0.086	**-0.567**	**-0.313**	0.160
	p-value	0.194	0.447	<.0001	0.005	0.156

^1^Pearson correlation coefficient with p-value testing that it is zero

The proposed algorithm was applied to the breast tumor data with 1000 bootstrapped (sampled with replacement) data sets of size 80 from 80 samples. For each possible gene subset size (1-6), Table 2 lists subsets with the lowest bootstrap upper 95% confidence bound for the variance of the log geometric mean. The absolute lowest bound for the geometric mean variance (shown in bold) is achieved by the combination of two genes, ACTB and SF3A1. Thus, according to criterion (A), ACTB and SF3A1 provide the optimal gene subset. Table 3 presents the top 10 gene subsets with lowest overall (regardless of the set size) average rank of geometric mean variance in 1000 bootstrap samples. The same subset of two genes, ACTB and SF3A1, comes up optimal using the criterion (C). Figure 1 shows the upper 95% confidence bound for Var(GM) vs. the average overall rank of the corresponding gene subset, visualizing the optimality of ACTB and SF3A1. The subset of three genes, ACTB, PUM1, and SF3A1 is very close to ACTB and SF3A1 with respect to criterion (A) but not with respect to criterion (C), which adds confidence in ACTB and SF3A1 as the optimal set of reference genes.

**Table 2:** Breast tumor data: Top ranked by set size bootstrap 95% upper confidence limit (UCL) for the variance and standard deviation of the log geometric mean (GM).

Set Size(*)	ACTB	GAPDH	MRPL19	PSMC4	PUM	SF3A1	95% UCL Var(GM)	95% UCL StdDev(GM)
1	1	0	0	0	0	0	0.407	0.638
**2**	**1**	**0**	**0**	**0**	**0**	**1**	**0.349**	**0.591**
3	1	0	0	0	1	1	0.356	0.596
4	1	1	0	0	1	1	0.398	0.631
5	1	1	0	1	1	1	0.429	0.655
6	1	1	1	1	1	1	0.465	0.682

(*) in the column with gene name, 1 indicates that the corresponding gene is included in the subset and 0 that it is not included.

**Table 3:** Breast tumor data: Ten gene subsets with the smallest mean overall ranks of the variance of the log geometric mean (GM).

Set Size(*)	ACTB	GAPDH	MRPL19	PSMC4	PUM	SF3A1	Mean rank of Var(GM)
**2**	**1**	**0**	**0**	**0**	**0**	**1**	**1.2**
3	1	0	0	0	1	1	2.0
2	1	0	0	0	1	0	3.2
2	0	0	0	0	1	1	4.7
4	1	0	0	1	1	1	6.1
1	1	0	0	0	0	0	6.4
3	1	0	0	1	0	1	8.1
1	0	0	0	0	0	1	8.8
4	1	0	1	0	1	1	9.2
4	1	1	0	0	1	1	10.3
3	1	0	1	0	0	1	10.6

(*) in the column with gene name, 1 indicates that the corresponding gene is included in the subset and 0 that it is not included.

In contrast, using the model in [9], the geometric mean of four genes, MRPL19, PUM1, PSMC4, and SF3A1, has the smallest estimated variability (the innate standard deviation = 0.1490). The geometric mean corresponding to three genes, MRPL19, PUM1, and PSMC4 (the innate standard deviation = 0.1494) yields just a small increase in standard deviation. Thus, MRPL19, PUM1, and PSMC4 may be considered an optimal subset using the approach in [9].

For direct comparison of results, the empirical variances of the geometric means of selected gene subsets were computed for the actual log geometric means based on the optimal subsets identified by the proposed selection method and methods [4] and [9]. Table 4 reports these geometric means variances for gene subsets of size from 2 to 4 since the size of the optimal subsets ranges from 2 for the new method to 4 for the method [9]. The optimal 4-gene subset using the method [4] is not reported in [9]. Note that for any subset size from 2 to 4, the geometric mean variance of the genes selected using the proposed method is smaller than for the other two methods. The geometric mean of two genes, ACTB and SF3A1, selected as an optimal subset using the proposed method, has the smallest variance among all subsets (in bold in Table 4), and the number of genes in this optimal subset is smaller than the number of genes in the optimal or nearly optimal subsets identified by using the approaches in [4] or [9].

**Table 4:** Breast tumor data: Variability of log geometric means based on optimal gene subsets identified by various methods

Set Size	Method	Optimal set	Variance logGM	Std Dev logGM
2	Szabo et al	MRPL19, PUM1	0.517	0.719
2	Vandes. et al	MRPL19, PSMC4	0.629	0.793
2	New	ACTB, SF3A1	**0.321**	**0.567**
3	Szabo et al	MRPL19, PUM1, PSMC4	0.531	0.729
3	Vandes. et al	MRPL19, PUM1, PSMC4	0.531	0.729
3	New	ACTB, SF3A1, PUM1	0.327	0.572
4	Szabo et al ^1^	MRPL19, PUM1, PSMC4, SF3A1	0.464	0.681
4	New	ACTB, SF3A1, PUM1, GAPDH	0.369	0.607

^1^Same results using either the method of Vandesompele et al [4] or Szabo et al [9]

### Results for neuroblastoma data

For 34 neuroblastoma samples, the proposed new algorithm yielded the smallest upper bound for the variance of the geometric mean of six genes, ACTB, B2M, GAPDH, HPRT1, TBP, and YWHAZ. However, the subsets of four genes, ACTB, B2M, GAPDH, and TBP have a negligibly higher upper bound (0.303 vs. 0.298, Table 5). For these data, the methods in [4,9] yielded the same results for any gene set size from 2 to 6. These approaches yielded the optimal subset of again six but not the same reference genes (GAPDH, HPRT1, SDHA, UBC, HMBS, YWHAZ). That is, only three genes, GAPDH, HPRT1, and YWHAZ, were common for two optimal subsets using criterion (A) and previous approaches. Table 6 presents the top 10 gene subsets with the lowest overall (regardless of the set size) average rank of geometric mean variance in 1000 bootstrap samples. In these data, using the criterion (C) we do not identify the same subsets as using (A) as optimal. The overall average lowest rank corresponds to another set of 6 genes (ACTB, B2M, GAPDH, RPL13A, TBP, and YWHAZ). However, the optimal sets of six genes by criterion (A) and (C) have 5 genes (ACTB, B2M, GAPDH, TBP, and YWHAZ) in common and differ only by inclusion of RPL13A or HPRT1. Respectively, only two genes, GAPDH and YWHAZ, are common for two optimal subsets using criterion (C) and previous approaches.

**Table 5:** Neuroblastoma data: Top ranked by set size bootstrap 95% upper confidence limit (UCL) for the variance and standard deviation of the log geometric mean (GM).

Set Size(*)	**AC**^**1**^	B2M	**GA**^**2**^	**HM**^**3**^	**HP**^**4**^	**RP**^**5**^	**SD**^**6**^	TBP	UBC	**YW**^**7**^	95% UCL Var(GM)	95% UCL StdDev(GM)
1	0	0	1	0	0	0	0	0	0	0	0.458	0.677
2	0	0	1	0	0	0	0	0	0	1	0.340	0.583
3	1	0	1	0	0	0	0	0	0	1	0.340	0.583
4	1	1	1	0	0	0	0	1	0	0	0.303	0.550
5	1	1	1	0	1	1	0	0	0	0	0.299	0.547
6	1	1	1	0	1	0	0	1	0	1	0.298	0.546
7	1	1	1	0	1	1	0	1	0	1	0.303	0.550
8	1	1	1	0	1	1	1	1	0	1	0.317	0.563
9	1	1	1	1	1	1	1	1	0	1	0.334	0.578
10	1	1	1	1	1	1	1	1	1	1	0.353	0.594

(*) in the column with gene name, 1 indicates that the corresponding gene is included in the subset and 0 that it is not included

^1^AC - ACTB; ^2^GA - GAPDH; ^3^HM - HMBS; ^4^HP - HPRT1; ^5^RP - RPL13A; ^6^SD - SDHA; ^7^YW - YWHAZ

**Table 6:** Neuroblastoma data: Ten gene subsets with the smallest mean overall ranks of the variance of the log geometric mean (GM).

Set Size(*)	**AC**^**1**^	B2M	**GA**^**2**^	**HM**^**3**^	**HP**^**4**^	**RP**^**5**^	**SD**^**6**^	TBP	UBC	**YW**^**7**^	Mean rank of Var(GM)
6	1	1	1	0	0	1	0	1	0	1	52.1
7	1	1	1	0	1	1	0	1	0	1	59.9
5	1	1	1	0	0	1	0	1	0	0	63.8
6	1	1	1	0	1	1	0	0	0	1	76.7
6	1	1	0	0	1	1	0	1	0	1	87.5
5	1	1	1	0	0	0	0	1	0	1	92.2
6	1	1	1	0	1	1	0	1	0	0	93.3
7	1	1	1	1	1	1	0	0	0	1	95.9
7	1	1	1	1	0	1	0	1	0	1	96.4
7	1	1	1	0	0	1	1	1	0	1	103.0

(*) in the column with gene name, 1 indicates that the corresponding gene is included in the subset and 0 that it is not included

^1^AC - ACTB; ^2^GA - GAPDH; ^3^HM - HMBS; ^4^HP - HPRT1; ^5^RP - RPL13A; ^6^SD - SDHA; ^7^YW - YWHAZ

Figure 2 indicates that the set of 7 genes that include all the genes selected using criteria (A) and (C) (ACTB, B2M, GAPDH, HPRT1, RPL13A, TBP, and YWHAZ) may be considered optimal with respect to both criteria (A) (0.005 difference from the minimum upper bound in Table 5) and (C) (second lowest average rank in Table 6). However, the advantage of addressing both criteria may not be worth the increase of the set size from 4 (ACTB, B2M, GAPDH, and TBP) to 7 by adding HPRT1, RPL13A, and YWHAZ. In this situation, criterion (B) with the acceptable upper limit of 0.55 on the standard deviation scale would yield the optimal set ACTB, B2M, GAPDH, and TBP.

Table 7 reports the geometric mean variances and corresponding standard deviations for empirical geometric means based on gene subsets of sizes from 2 to 6 selected using the new approach (gene subsets reported in Table 5) and previously proposed approaches. Again, for any subset size from 2 to 6, the geometric mean variance of the genes selected using the proposed method is smaller than for the other two methods. Furthermore, even though previous and new approaches selected an optimal subset of six genes, the subset selected ignoring the correlation (GAPDH, HPRT, SDHA, UBC, HMBS, YWHAZ) has 18% higher standard deviation than the subset selected using the proposed approach (ACTB, GAPDH, B2M, HPRT1, TBP, YWHAZ). Also, the optimal subsets of size 4, 5, and 6 selected by the new approach have virtually the same standard deviation of the actual geometric means. Hence by selecting the optimal subset of 4 genes (ACTB, B2M, GAPDH, and TBP) one may expect ~15% reduction in the standard deviation of the normalizing factors and a smaller number of genes (4 vs. 6) to be processed for each sample.

**Table 7:** Neuroblastoma data: Variability of log geometric means based on optimal gene subsets identified by various methods

Set Size	Method	Optimal set	Variance logGM	Std Dev logGM
2	Vand	GAPDH, HPRT	0.327	0.572
2	Szabo	GAPDH, SDHA	0.374	0.612
2	New	GAPDH, YWHAZ	0.250	0.500
3	Old^1^	GAPDH, HPRT, SDHA	0.348	0.590
3	New	ACTB, GAPDH, YWHAZ	0.255	0.505
4	Old^1^	GAPDH, HPRT, SDHA, UBC	0.361	0.601
4	New	ACTB, B2M, GAPDH, TBP	0.231	**0.480**
5	Old^1^	GAPDH, HPRT, SDHA, UBC, HMBS	0.358	0.598
5	New	ACTB, B2M, GAPDH, HPRT1, RPL13A	0.224	**0.473**
6	Old^1^	GAPDH, HPRT, SDHA, UBC, HMBS, YWHAZ	0.319	0.565
6	New	ACTB, GAPDH, B2M, HPRT1, TBP, YWHAZ	0.227	**0.477**

^1^Same results using either the method of Vandesompele et al [4] or Szabo et al [9]

### Results for five reference genes for GUCY2C in blood

For five candidate reference genes for GUCY2C (ACTB, GAPDH, HPRT, PPIB, and TFRC), the new approach was applied to the log transformed relative expression levels for direct comparison with previously proposed methods and to the threshold cycle (Ct) numbers because Ct numbers are actually used for efficiency adjusted relative quantification [15].

**Figure 4:**
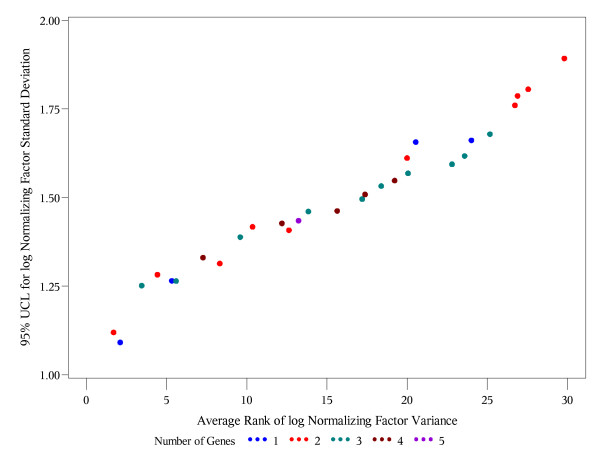
**Blood data: 95% UCL vs. the average overall rank of the normalizing factors (expression levels)**. Each point represents one of the possible 33 = 2^5^-1 gene subsets. Different colors are used for the subsets with different numbers of genes included. The x-coordinate is the average overall rank of the corresponding normalizing factor variance. The y-coordinate is the upper 95% confidence limit (95% UCL) for the standard deviation of the log normalizing factor.

Tables 8 and 9 shows the subsets of each possible size (1-5) with the lowest bootstrap upper 95% confidence bound for the variance of the log geometric mean and corresponding standard deviations based on the log transformed relative expression levels and Ct numbers, respectively. For all set sizes except 4, the same gene subsets are selected using relative expression levels and Ct numbers. However, the smallest upper 95% confidence bound is achieved by a single gene (GAPDH) if we use the relative expression levels (Table 8), and by two genes (GAPDH and TFRC) using the Ct numbers. Tables 10 and 11 present the top 10 gene subsets with lowest overall (regardless of the set size) average rank (in 1000 bootstrap samples) of geometric mean variance based on the log transformed relative expression levels and Ct numbers, respectively. Notably, GAPDH and TFRC have the lowest average rank under both conditions. Hence, this set of 2 genes is optimal with respect to both criteria (A) and (C) if we use the Ct numbers (see Figure 3). Figure 4 shows that single GAPDH and the subset GAPDH and TFRC are very close in terms of both criteria (A) and (C) if we use the log transformed relative expression levels. These results suggest that the use of relative expression levels may alter the correlation pattern among the candidate reference genes, and if Ct numbers are used for relative quantification, then selection of the reference genes should utilize the Ct numbers as well.

**Table 8:** Blood data: Top ranked by set size bootstrap 95% upper confidence limit (UCL) for the variance and standard deviation of the log geometric mean (GM) based on log transformed relative expression levels.

Set Size(*)	ACTB	GAPDH	HPRT1	PPIB	TFRC	95% UCL Var(GM)	95% UCL StdDev(GM)
1	0	1	0	0	0	**1.19**	1.09
2	0	1	0	0	1	1.25	1.12
3	0	1	1	0	1	1.57	1.25
4	0	1	1	1	1	1.77	1.33
5	1	1	1	1	1	2.06	1.43

(*) in the column with gene name, 1 indicates that the corresponding gene is included in the subset and 0 that it is not included.

**Table 9:** Blood data: Top ranked by set size bootstrap 95% upper confidence limit (UCL) for the variance and standard deviation of the log geometric mean (GM) based on Ct numbers.

Set Size(*)	ACTB	GAPDH	HPRT1	PPIB	TFRC	95% UCL Var(GM)	95% UCL StdDev(GM)
1	0	1	0	0	0	6.22	2.49
2	0	1	0	0	1	6.06	2.46
3	0	1	1	0	1	6.66	2.58
4	1	1	1	0	1	7.29	2.70
5	1	1	1	1	1	7.91	2.81

(*) in the column with gene name, 1 indicates that the corresponding gene is included in the subset and 0 that it is not included.

**Table 10:** Blood data: Ten gene subsets with the smallest mean overall ranks of the variance of the log geometric mean (GM) based on log transformed relative expression levels.

Set Size(*)	ACTB	GAPDH	HPRT1	PPIB	TFRC	Mean rank of Var(GM)
2	0	1	0	0	1	1.5
1	0	1	0	0	0	2.8
3	0	1	1	0	1	3.7
3	1	1	0	0	1	5.3
2	0	1	1	0	0	5.7
1	0	0	0	0	1	7.0
4	1	1	1	0	1	8.1
3	0	1	0	1	1	8.2
2	1	1	0	0	0	8.8
4	0	1	1	1	1	10.7

(*) in the column with gene name, 1 indicates that the corresponding gene is included

in the subset and 0 that it is not included.

**Table 11:** Blood data: Ten gene subsets with the smallest mean overall ranks of the variance of the log geometric mean (GM) based on Ct numbers.

Set Size(*)	ACTB	GAPDH	HPRT1	PPIB	TFRC	Mean rank of Var(GM)
2	0	1	0	0	1	1.7
1	0	1	0	0	0	2.1
3	0	1	1	0	1	3.5
2	0	1	1	0	0	4.4
1	0	0	0	0	1	5.3
3	0	1	0	1	1	5.6
4	0	1	1	1	1	7.3
2	0	1	0	1	0	8.3
3	0	1	1	1	0	9.6
2	0	0	1	0	1	10.4

(*) in the column with gene name, 1 indicates that the corresponding gene is included in the subset and 0 that it is not included.

For comparison, model 1a in [9] was also fitted treating the sample effects as fixed and assuming that the correlation is zero. Based on this model, the estimated variances of the reference genes were ordered as 0.110 (TFRC), 0.377 (GAPDH), 0.400 (PPIB), 0.441 (HPRT), 3.059 (ACTB). The variances of corresponding the geometric means were 0.122 (geometric mean of TFRC, GAPDH), 0.099 (geometric mean of TFRC, GAPDH, PPIB), 0.083 (geometric mean of TFRC, GAPDH, PPIB, HPRT), 0.176 (geometric mean of TFRC, GAPDH, PPIB, HPRT, ACTB). This implies the optimal subset of 4 genes, GAPDH, TFRC, PPIB, and HPRT. Finally, the variability of empirical log geometric means based relative expression levels on was computed (Table 12) based on only the subsets of genes selected. The smallest variability of log geometric means was achieved again by GAPDH and TFRC, which were selected as an optimal subset of size 2 using the approach in [9] but not in [4]. However, the Szabo et al [9] approach picked the 4-gene subset as the overall best one. Thus, using the proposed approach allows reducing the optimal number of genes required for normalization by half while also reducing the variability of the normalizing factor.

**Table 12:** Blood data: Variability of log geometric means based on optimal gene subsets identified by various methods

Set Size	Method	Optimal set	Variance logGM	Std Dev logGM
2	Szabo et al	TFRC, GAPDH	0.98	0.99
2	Vandes. et al	TFRC, HPRT	1.47	1.21
2	New	TFRC, GAPDH	**0.98**	0.99
3	Szabo et al	TFRC, GAPDH, PPIB	1.26	1.12
3	Vandes. et al	TFRC, GAPDH, PPIB	1.62	1.27
3	New	TFRC, GAPDH, HPRT	1.16	1.08
4	All methods	GAPDH, PPIBA, TFRC, HPRT	1.34	1.16

### Simulation study

A small simulation study was conducted to evaluate the performance of the proposed approach assuming varying degrees of innate correlation among reference genes, independent of the variance component corresponding to the sample random effect. Samples of size 25, 40, or 80 of 5-dimensional vectors, representing log transformed expression levels, were generated from the 5-variate normal distribution according to model (4). Since the mean part of the model does not affect either the new or previously proposed methods, without loss of generality, it was assumed that the mean vector had all components equal to zero (**g **= **0**). The covariance matrix **V **of the simulated 5-variate normal samples had the structure **V **= σ^2^1_J×J _+ **R**, where σ^2 ^is the variance component for sample random effect and **R **is the covariance matrix of random effects of genes, and J = 5. Table 13 describes the correlation structures and resulting matrices **V **used in five different simulation scenarios. The values of σ, the standard deviation for the sample random effect, ranged from 0.02 to 0.16, while correlation coefficients corresponding to the **R **matrices, were 0, ±0.2, or ±0.4, representing zero, weak and strong correlation respectively. The **R **matrices used were defined by five standard deviations, corresponding to the innate variances of the each gene and by the correlation matrix shown. The values of the standard deviations were chosen so that resulting elements of matrices **V **were similar in magnitude to the estimates from the real data examples. Table 13 provides the true minimum variances of the subset means for each subset size, computed using (6) and true assumed matrix **V**. The values in bold correspond to the absolute minimum variance of the mean for any possible subset size and the size of that optimal subset. Table 13 also gives the corresponding variances using the approach in Szabo et al [9] that is, estimating **V **as a diagonal matrix and assuming the sample effect to be fixed rather than random. The corresponding absolute minimum variance of the mean for any possible subset size is shown in bold italic. Since the results using the method of Szabo et al [9] were generally very consistent with the approach of Vandesompele et al [4], in this simulation study, the proposed approach was compared only to the approach in Szabo et al [9].

**Table 13:** Design of the simulation study

													**Min Var of NF**^**1**^	
													
Scenario	Std Dev	Correlation Matrix of R					Total Covariance Matrix V					No Genes	True	**Uncorr**^**2**^
	0.30	1	0	0	0	0	0.25	0.16	0.16	0.16	0.16	1	0.250	0.250
Uncorrelated R	0.35	0	1	0	0	0	0.16	0.28	0.16	0.16	0.16	**2**	**0.213**	***0.133***
Sample Random	0.80	0	0	1	0	0	0.16	0.16	0.80	0.16	0.16	3	0.255	0.148
Effect Var = 0.16	0.90	0	0	0	1	0	0.16	0.16	0.16	0.97	0.16	4	0.264	0.144
	1.00	0	0	0	0	1	0.16	0.16	0.16	0.16	1.16	5	0.267	0.139

	0.60	1	0.2	0.2	0.2	0.2	0.38	0.10	0.11	0.15	0.16	1	0.380	0.380
Corr Coef = 0.2	0.70	0.2	1	0.2	0.2	0.2	0.10	0.51	0.13	0.17	0.19	2	0.275	0.223
Sample Random	0.75	0.2	0.2	1	0.2	0.2	0.11	0.13	0.58	0.19	0.20	**3**	**0.239**	***0.164***
Effect Var = 0.02	1.10	0.2	0.2	0.2	1	0.2	0.15	0.17	0.19	1.23	0.28	4	0.275	0.169
	1.20	0.2	0.2	0.2	0.2	1	0.16	0.19	0.20	0.28	1.46	5	0.301	0.167

	0.42	1	-0.2	-0.2	0.2	0.2	0.28	0.06	0.06	0.15	0.15	1	0.276	0.276
Corr Coef = ±0.2	0.45	-0.2	1	-0.2	0.2	0.2	0.06	0.30	0.06	0.15	0.15	2	0.176	0.145
Sample Random	0.48	-0.2	-0.2	1	0.2	0.2	0.06	0.06	0.33	0.16	0.16	**3**	**0.141**	0.101
Effect Var = 0.1	0.60	0.2	0.2	0.2	1	0.2	0.15	0.15	0.16	0.46	0.17	4	0.166	0.086
	0.60	0.2	0.2	0.2	0.2	1	0.15	0.15	0.16	0.17	0.46	5	0.175	***0.073***

	0.30	1	-0.4	0.0	0.0	0.0	0.25	0.11	0.16	0.16	0.16	1	0.250	0.090
Corr Coef = ±0.4	0.40	-0.4	1	0.0	0.0	0.0	0.11	0.32	0.16	0.16	0.16	**2**	**0.199**	***0.063***
Sample Random	0.60	0.0	0.0	1	0.0	0.0	0.16	0.16	0.52	0.16	0.16	3	0.217	0.068
Effect Var = 0.16	0.70	0.0	0.0	0.0	1	0.4	0.16	0.16	0.16	0.65	0.38	4	0.223	0.069
	0.80	0.0	0.0	0.0	0.4	1	0.16	0.16	0.16	0.38	0.80	5	0.244	0.070

	0.40	1	0.4	0.4	0.4	0.4	0.26	0.18	0.21	0.23	0.24	1	0.260	0.260
Corr Coef = 0.4	0.50	0.4	1	0.4	0.4	0.4	0.18	0.35	0.24	0.26	0.28	**2**	**0.243**	0.153
Sample Random	0.70	0.4	0.4	1	0.4	0.4	0.21	0.24	0.59	0.32	0.35	3	0.274	0.133
Effect Var = 0.1	0.80	0.4	0.4	0.4	1	0.4	0.23	0.26	0.32	0.74	0.39	4	0.302	0.121
	0.90	0.4	0.4	0.4	0.4	1	0.24	0.28	0.35	0.39	0.91	5	0.331	***0.114***

^1^NF - normalizing factor

^2^Assuming Szabo et al [9] model

The results of the simulation study are summarized in terms of sensitivity to identifying the optimal subset with the absolute minimum variance of the mean. Table 14 gives the percentage of simulated data sets, for which the corresponding method (proposed criterion **A **(UCL), proposed criterion **B **(Rank), and method in [9]) correctly identified the optimal subset of genes. This percentage may also be interpreted as the power of the corresponding procedure to detect the optimal subset. For each scenario and sample size (25, 40, or 80), 400 data sets were simulated to have 95% confidence interval for sensitivity of width <0.1 (±5%).

**Table 14:** Results of the simulation study

		Sensitivity to optimal subset		
		
Scenario	No of samples	**UCL**^**1**^	**Rank**^**2**^	**Szabo**^**3**^
Uncorrelated R	25	43.00	41.75	60.25
Sample Random	40	53.50	53.25	73.50
Effect Var = 0.16	80	81.25	81.50	86.25

All Corr Coef = 0.2	25	34.25	36.50	38.75
Sample Random	40	53.25	55.25	46.25
Effect Var = 0.02	80	75.75	74.50	57.25

Corr Coef = ±0.2	25	48.50	55.00	0.00
Sample Random	40	68.00	72.75	0.25
Effect Var = 0.1	80	91.50	93.50	0.00

Corr Coef = ±0.4	25	36.50	31.50	8.50
Sample Random	40	49.25	42.75	7.25
Effect Var = 0.16	80	63.50	60.25	3.75

All Corr Coef = 0.4	25	37.5	40.8	23.3
Sample Random	40	49.8	51.3	21.0
Effect Var = 0.1	80	68.0	71.5	21.3

^1^Criterion (**A**) (minimum 95% upper confidence limit for standard deviation of the normalizing factor)

^2^Criterion (**C**) (minimum average rank of the normalizing factor variance)

^3^Minimum standard deviation of the normalizing factor variance as in Szabo et al [9]

The results of our simulation study suggest that for truly uncorrelated candidate reference genes, the proposed approach may have lower power/sensitivity than the method of Szabo et al [9]. This may be expected since the true **V **has the structure as assumed in [9], while our approach would estimate unnecessary extra parameters in unstructured **V**. For equally weakly positively correlated candidate reference genes, performance of our and approach in [9] was similar for smaller sample sizes (25-40), while the new proposed approaches were better for N = 80. When the same weak correlation was assumed positive for some pairs of genes and negative for others, then the proposed approach was clearly superior to the method in [9]. Similarly, our approach performed much better in the scenario with some strongly positively, some strongly negatively, and some uncorrelated pairs of candidate reference genes. Finally, in the case of equally strongly positively correlated candidate reference genes, we also observed an advantage of the proposed approach.

## Discussion

In this work, we developed an approach for selecting an optimal set of reference genes for normalization in RT-PCR. The key difference from previously proposed methods is that assumption of independence among candidate reference genes is relaxed, and, instead, the estimated correlation among the genes is incorporated into estimates of variability of the prospective normalizing factors. The proposed approach does not explicitly estimate correlation among the genes, but implicitly the correlation is incorporated into the estimate of the total covariance matrix **V**. Then the variance of a log transformed prospective normalizing factor is estimated by substituting the estimated **V **into (6).

To overcome uncertainty in estimating a large number of covariance parameters from usually small data sets, we employ bootstrap to obtain robust upper confidence bounds for the variance of the log geometric means of multiple genes. These bounds allow comparing various gene subsets as prospective normalizing factors, but also may be used in sample size calculations while designing an RT-PCR study. Our approach also allows certain flexibility to choose a criterion for selecting the optimal subset(s) of the reference genes unless one subset meets all the criteria.

Here, our primary focus was on selecting reference genes for normalizing target gene expressions from one tissue as motivated by the study of guanylyl cyclase C (GUCY2C) mRNA expression in blood. Our methodology is easily extendable to multiple tissues or inter-species comparisons by incorporating fixed effects for between-tissue or between-species differences into the mean sub-model **Aβ **in (3), as long as one can assume that variances and correlation among the genes do not change between tissues or between species. If they do change between tissues or between species, then selecting the same reference genes for different tissues or different species may not be appropriate, or careful consideration may be required to set appropriate criteria of optimal properties of the reference genes that may behave differently in different tissues or species.

In the considered data examples, the use of the proposed methodology yielded generally smaller optimal subsets of the reference genes with smaller variability of the normalizing factors. In direct comparisons, the normalizing factor variances (based on the genes from the selected subset only) were reduced by 27-32% when using the proposed selection approach instead of the methods [4] and [9]. Taken together, the smaller number of reference genes and smaller normalizing factors could result in cost savings due to both reduced primer and probe needs and potentially smaller numbers of samples required for the experiment overall.

## Conclusions

The proposed approach performs comprehensive and robust evaluation of the variability of normalizing factors based on all possible subsets of candidate reference genes rather than addressing the stability of individual reference genes. The results of this evaluation provide flexibility to choose more important criterion for selecting the optimal subset(s) of the reference genes unless one subset meets all the criteria. This new approach identifies gene subset(s) with smaller variability of normalizing factors than current standard approaches when there is some nontrivial innate correlation among the candidate genes.

## Authors' contributions

SAW and SS initiated the biological problem. CW, IC, SS, SAW, and TH designed the validation study of five candidate reference genes for normalization of guanylyl cyclase C (GUCY2C). IC and TH designed the statistical methods and analyses. CW conducted the RT-PCR experiments. IC and YL conducted the analysis, devised algorithms and wrote the computer programs. SC carried out the simulation study and prepared the figures. All authors have read and approved the final manuscript.

## Supplementary Material

Additional file 1 SAS program file with the code implementing the proposed algorithm. Click here for file
